# On the optimistic performance evaluation of newly introduced bioinformatic methods

**DOI:** 10.1186/s13059-021-02365-4

**Published:** 2021-05-11

**Authors:** Stefan Buchka, Alexander Hapfelmeier, Paul P. Gardner, Rory Wilson, Anne-Laure Boulesteix

**Affiliations:** 1 Institute for Medical Information Processing, Biometry and Epidemiology, LMU, Munich, Germany; 2 Institute of Medical Informatics, Statistics and Epidemiology, School of Medicine, TUM, Munich, Germany; 3 Institute of General Practice and Health Services Research, School of Medicine, TUM, Munich, Germany; 4 Department of Biochemistry, University of Otago, Otago, New Zealand; 5 Research Unit Molecular Epidemiology, Institute of Epidemiology, Helmholtz Zentrum München, German Research Center for Environmental Health, Neuherberg, Germany

**Keywords:** Benchmarking, Optimistic bias, Neutral comparison study, Illumina HumanMethylation450K BeadChip, Normalization

## Abstract

Most research articles presenting new data analysis methods claim that “the new method performs better than existing methods,” but the veracity of such statements is questionable. Our manuscript discusses and illustrates consequences of the optimistic bias occurring during the evaluation of novel data analysis methods, that is, all biases resulting from, for example, selection of datasets or competing methods, better ability to fix bugs in a preferred method, and selective reporting of method variants. We quantitatively investigate this bias using an example from epigenetic analysis: normalization methods for data generated by the Illumina HumanMethylation450K BeadChip microarray.

**Supplementary Information:**

The online version contains supplementary material available at (10.1186/s13059-021-02365-4).

## Background

Many studies in the biomedical sciences employ computational methods to process and evaluate data in order to answer the research question presented. Sometimes these methods are relatively simple, such as *t*-tests to detect differentially expressed genes, but in many cases the methods are complex, and there is no final “gold standard” as to which method to apply in a given setting. Further complicating matters is that data acquisition, data structure, and research questions themselves evolve quickly. As such, the development of new computational methods is an active field of research, a situation which constantly introduces new methods into the literature, often in a comparison with existing methods. Yet despite the frequency of these types of papers, there is a surprising lack of guidance on the appropriate design and reporting of studies presenting and evaluating new computational methods [1, 2]. It is not clear how new methods and their performances should be described and how studies comparing performances of methods should be designed. This absence of guidance is in strong contrast to the intensive decades-long discussions in the biomedical literature on the design of other types of health science studies and the numerous resultant reporting guidelines and guidance documents developed, such as those for observational studies [3], randomized clinical trials [4], and meta-analyses [5].

The abundance of literature introducing computational methods is often perceived as confusing, and data analysts have difficulty keeping pace with methodological developments in their field and thus choosing the most appropriate method for their data and question at hand [6]. In this context, benchmarking studies (i.e., systematic studies comparing the behaviors and performances of computational methods) [7, 8], especially those that are neutral [9], and those that incorporate a blinding procedure [10], community computational challenges, and meta-analyses of benchmarking studies [11] (i.e., studies combining the results of several benchmark studies) are crucial in providing guidance for the methods’ potential users [12].

A major issue related to the design and reporting of studies introducing new computational methods is optimistic bias: the performance of a newly introduced method is often—intentionally or unintentionally—oversold. Though little acknowledged, publication bias likely pervades the computational literature [13]. In fear of not getting published, researchers may feel pressure to report the superiority of their new method, succumbing to the temptations of selective reporting or data dredging, practices which can lead to significant optimistic bias with regard to the performance of their new method, a consequence illustrated in several empirical studies on classification using high-dimensional data [14, 15]. The term “self-assessment trap” has also been used to describe these mechanisms [16, 17].

For example, authors could preferentially report the results for datasets [14] or performance metrics [16] for which their new method works better than for existing ones. They may be more attentive to—and proficient in—choosing parameters or fixing bugs for their method than for competing methods. For example, if a new method yields a noticeably bad result in one analysis, the authors are likely to look for a potential bug, while they would accept the bad result—without further investigation—if observed for a competing method. Finally, if they develop several variants of their method and report only the variant that (perhaps by chance) performs best on the datasets and metrics used as examples, this variant will likely perform worse on future datasets, i.e., the good performance does not generalize. And although this bias weakens with a greater number of datasets used for fitting and evaluation, another problem may be that authors test their method on datasets from contexts with which they are familiar, and failing to outline this bias in the limitations. Previous studies have indicated that performance evaluation can highly depend on the type of datasets examined [18], a source of variation perhaps revealed in later studies involving types of datasets unanticipated by the original method’s authors.

A consequence of the conscious and unconscious decisions biasing a study introducing a new method is that subsequent comparison studies by independent authors, “neutral” studies, often fail to replicate the superior performance of the method in question [19]. We assessed this bias quantitatively for a class of computational methods, namely methods to preprocess the raw data produced by the HumanMethylation450K BeadChip (450K), a microarray used to detect the methylation levels of approximately 450,000 genetic loci [20] (many of these methods having been modified to be applicable to the current EPIC BeadChip). Data preprocessing can crucially affect later data analysis [21, 22] in the context of epigenome-wide studies. Dozens of 450K preprocessing methods have been proposed in recent years, along with extensive comparison analyses [22], both in papers introducing new methods and in neutral benchmark studies, an ideal situation for investigation into the potential optimistic bias discussed here.

## Results and discussion

**Fig. 1:**
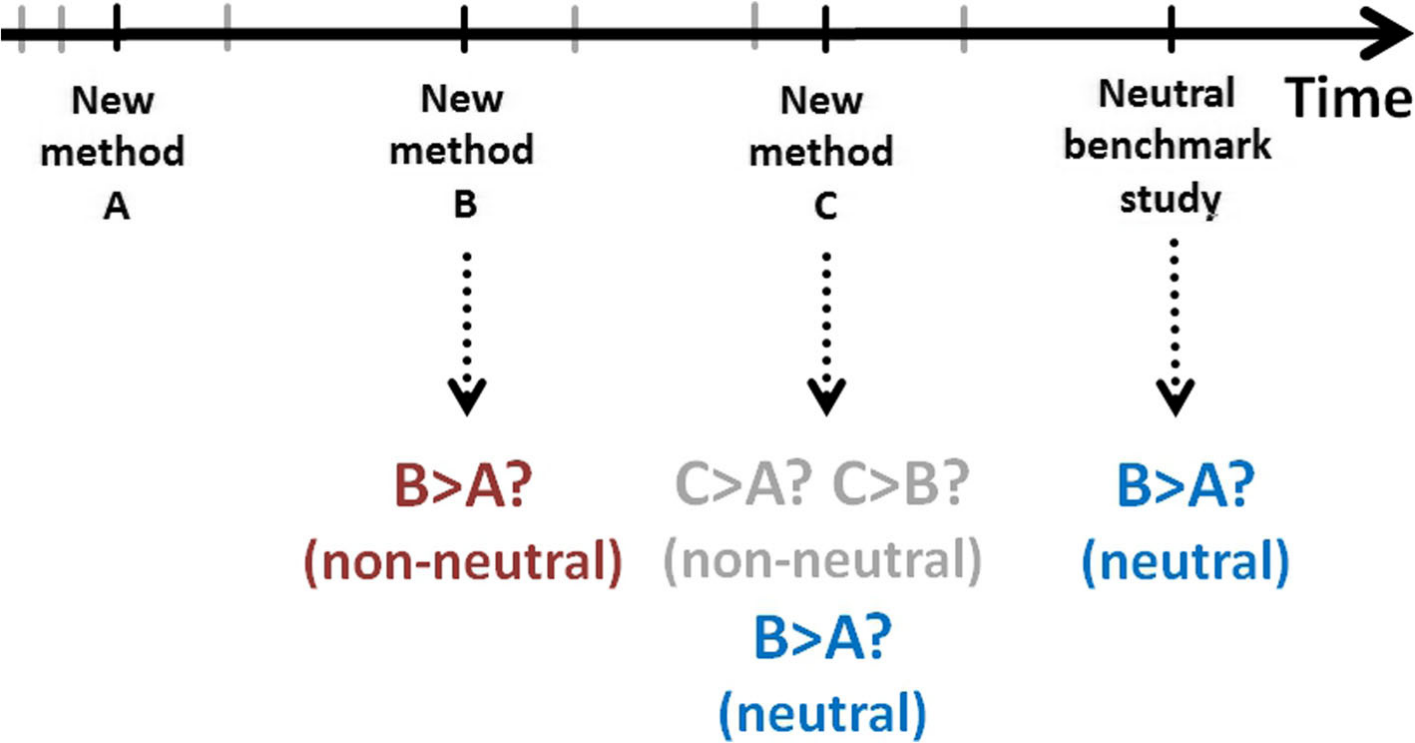
We examine the point in time where a method “A” exists in the literature, and a new method “B” is introduced. In the paper presenting method B, the authors compare it to method A: a “non-neutral comparison” (dark red color, see also Fig. 2), as method B’s authors may have some bias in presenting the results. Some time later, a paper introducing another method, “C”, is published. The authors compare C to existing methods A and B, which implies also a comparison of B to A, although this comparison is not the study’s focus. This latter study is assumed to be neutral (dark blue color, see also Fig. 2) with respect to A and B and is termed a “neutral benchmark study”

We surveyed papers presenting new methods and checked whether these new methods were assessed as better than existing competitors. We then investigated whether the same pairwise comparisons, when performed in later—presumably unbiased—studies, again evaluated the new method as better than the old. This principle is schematically illustrated in Fig. 1. Imagine that a method “B” is introduced in the literature some time after a method “A” had been introduced. In the paper presenting method B, the authors compare it to method A (typically, they find that B is better than A). This is what we call a non-neutral comparison because the authors are interested in demonstrating the superiority of method B. Some time later, a paper suggesting another method, “C”, is published. The authors compare C to existing methods A and B, which implies that this study also compares B to A, although this latter comparison is not the paper’s focus. This study is assumed to be neutral with respect to the comparison of A and B, exactly as the later study termed a “neutral benchmark study” in Fig. 1.

**Fig. 2:**
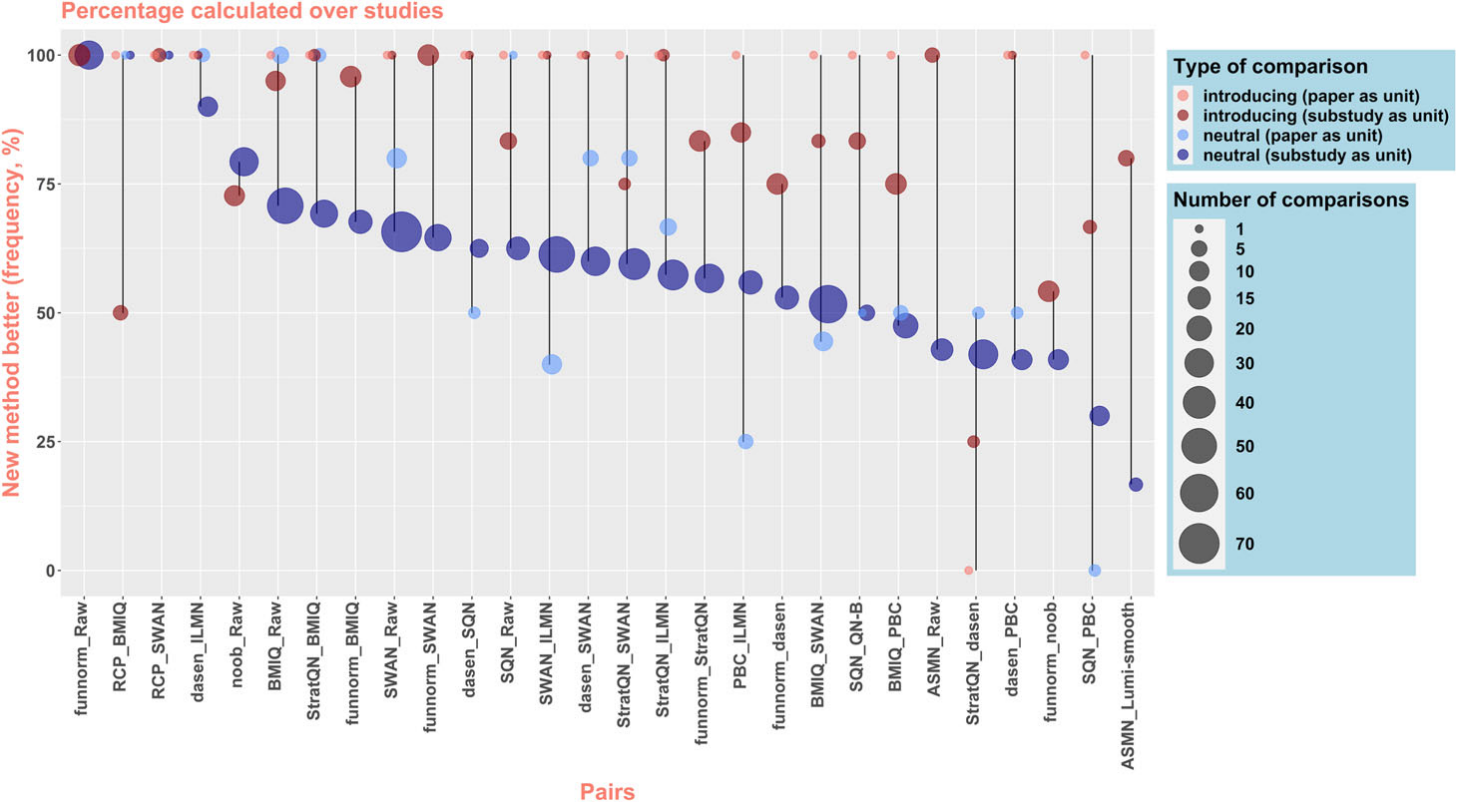
Comparisons between 450K data normalization methods: percentages of comparisons identifying the newer method as better than the older. The *x*-axis shows which two methods are being compared (see Additional files 1 and 2 for the references of the methods, abbreviations, and any aliases used in the paper). “Introducing” (i.e., “non-neutral”) comparisons (red dots) are those from publications introducing the new method. “Neutral” comparisons (blue dots) are from subsequent studies written by authors who developed neither of the methods being compared. Two analyses are presented. In the first analysis (light dots), the percentage is calculated over papers, i.e., where the overall rank from a paper is taken as the comparison. The percentage is thus either 0% or 100% for introducing comparisons, as for each pair there is only one introducing study (that in which the newer method is introduced). Papers were excluded from this analysis if individual components of the evaluation used only a subset of the methods examined in the paper (“partial substudies”). In the second analysis (dark dots), the percentage is calculated over substudies, i.e., individual comparisons within a paper. The “number of comparisons” refers to the number of papers (for the first analysis, which takes papers as units) or to the number of substudies (for the second analysis, which takes substudies as units)

In the simplified scenario of Fig. 1, we have one non-neutral study and two neutral studies, all comparing A and B. In the terminology commonly used in the debate on the replication crisis, the former could be denoted as an “original study” and the latter two as “replication studies”. Of course, in general, there may be any number of neutral studies, not just two. Most importantly, in our survey, we did not focus on a specific pair of methods, but looked for all pairs that were compared in the non-neutral study introducing the newer one and at least one subsequent neutral study.

Note that each pair of methods thus acts as its own control, helping to avoid problems due to confounding, such as through chronological time effects (if scientific progress works correctly, new methods are expected to be on average better than older ones, although this is not necessarily the case in practice [23]).

We identified 27 relevant studies (see supplement for details). We extracted pairs of methods that were compared in the non-neutral study introducing the newer of the two and at least one subsequent study which was neutral with respect to this pair (i.e., a paper introducing a third method or a neutral study not introducing a new method).

Some of the papers present several substudies (typically investigating different aspects of the methods), each comparing all or a subset of the methods considered in the paper. In a *first analysis*, we focused on the substudies that compare all the methods considered in the paper, i.e., we excluded the “partial substudies.” With this strategy, we found 19 pairs of methods compared both in the paper introducing the newer of the two and in at least one subsequent paper which was neutral with respect to this pair. For each pair and each paper, we recorded whether the newer method was ranked better than the older method. In the *second analysis*, we considered substudies as independent studies, i.e., ignored that they exist in “clusters” (the paper they come from), and did not exclude incomplete substudies. This yielded a total of 28 pairs. For each pair and each substudy, we again recorded whether the newer method was ranked better than the older.

The supplement provides full details on the analysis methods, and the data and R code to generate the results.

The results are displayed in Fig. 2. In the first analysis (considering papers as unit), the new method was ranked better than the older in 94.7% (18 of 19) of the comparisons from non-neutral papers introducing the new method, a very high rate similar to one detailed in a previous survey of the statistical and bioinformatics literature [9]. In neutral comparisons (subsequent papers that were neutral with respect to the considered pair), these same new methods outperformed 64.3% of their paired competitors (49.5 of 77 neutral comparisons, where pairs with equal performances count as 0.5). This rate lies between the rate for non-neutral papers and the rate of 50% assumed for a method that performs equally well as other methods. This finding suggests a noteworthy optimistic bias in favor of new methods in the papers introducing them, but also the realization of scientific progress, i.e., newer methods are on average superior to older.

The second analysis (considering substudies as unit) shows the same trends. The newer method was ranked better than the older in 83.2% (136.5 of 164) of the comparisons from non-neutral substudies, revealing that even according to biased authors, their method is not superior in every situation. This rate is once more higher than the 61.2% (408.5 of 667) observed for neutral substudies, again suggesting optimistic bias. It is however much lower than the 94.7% observed in the first analysis, which is in agreement with Norel et al.’s claim that “when the number of performance metrics is larger than two, most methods fail to be the best in all categories assessed” [16]: even the overall “better” method, as judged by the first analysis, is recognized by the authors of the better method as performing worse in some substudies.

Our study has some limitations. We neither performed a systematic literature search nor assessed the quality of the investigated papers, in particular the quality of the performance measures, as our study is meant as illustrative. The evaluator extracting the data could obviously not be blinded to the type of paper (non-neutral or neutral). This lack of blindness could have slightly distorted our results, as the evaluator’s expectation was that new methods would tend to be optimistically rated in the papers introducing them. This expectation might have affected, for example, his (partly subjective) evaluation of blurred or ambiguous graphical results within the papers being evaluated. Moreover, not taken into account in our study were the sizes of the differences between method performances: a method was considered either better or worse than the other, which obviously leads to a loss of information and precision.

The precise definition of a “method” also presents a problem. Many of the papers here evaluate the methods within a full “pipeline” of preprocessing, with such optional steps as background correction and elimination of probes based on detection *p*-values: the result is that comparisons between two methods in different papers may be based on different pipelines (it should however be noted that the availability of such pipeline “parameters” presents another opportunity for preferential reporting). In a similar vein, we were required to make subjective decisions on whether different implementations of similar algorithms constituted distinct or equal methods. Similarly, the evolution of a method over time was also not taken into account. Authors often release new (hopefully improved) versions of packages implementing their methods; when two methods are compared and then subsequently compared at a later time, the evaluations may not be completely consistent, although likely lacking systematic bias.

Truly “neutral” authorship could also not be verified, as we are ignorant of any personal feelings and connections our neutral-labelled authors may have, and extensive authorship lists may have overlap we did not take into account. Most importantly, the interpretation of the complex, multidimensional comparison of methods from the papers was very difficult. In particular, due to dependence patterns (within studies and between methods), standard statistical inference (e.g., deriving confidence intervals for the above-mentioned rates) was impossible. Despite these limitations, we feel this study convincingly illustrates the issue of over-optimism and indicates that its order of magnitude is not negligible.

## Solutions?

These observations highlight a disturbing issue: it is likely the results with regard to new methods presented in the computational literature are considerably biased in favor of the new method, and weaknesses of these new methods tend to be ignored. These biases and omissions are a problem for readers who rely on this literature to select methods. This situation calls for solutions at different levels of the scientific publishing process.

In an ideal world, authors would report the performance of their new methods in a balanced and transparent manner. They would not cherry-pick their best results, for example across a large set of results obtained through different configurations of datasets, simulation scenarios, parameter settings, or performance metrics, while sweeping the other results—those making the new method look less impressive—under the carpet. To achieve this long-term goal, journal editors and reviewers have a major responsibility. For authors to feel comfortable reporting balanced results and detailing the weaknesses of their new methods, the acceptance of nuanced pictures and open statements must increase. Editors and reviewers should become more tolerant towards methods that are not reported to perform best universally: although “groundbreaking” discoveries occasionally occur—i.e., a new method outperforms those existing in all respects—such a scenario is unrealistic, and the expectation of such results engenders malpractice.

Even in the ideal world described above, however, bias in favor of the new method cannot be completely eliminated, for example because the authors are more familiar with their new method than with competing methods. Moreover, a significant move towards this ideal world cannot realistically be achieved quickly. For these reasons, we believe that users of methods would strongly benefit from more high-quality neutral comparison studies, which tend to be more reliable than studies introducing a new method. Here the general scientific community and the journals in particular can play a positive role by acknowledging that neutral method-comparison studies are valuable research contributions—as some journals including *Genome Biology* [12] have already started to do in the last few years. This acknowledgment would relax the pressure on scientists to constantly produce new methods, freeing them to objectively investigate the method—new or existing—most appropriate to their data and research questions. That said, benchmark studies alone are not enough. Users of methods should be educated in the proper interpretation of benchmark studies: selecting methods requires expertise, and benchmark studies give a limited picture of the situation in a specific context perhaps not relevant to the given reader. For example, the choice of appropriate performance measures is not easy; in the context of methylation data analysis, see the case of evaluation criteria related to the reduction of technical variation, which are widely used but of questionable relevance [24].

More generally, researchers conducting benchmark studies have to make a myriad of design choices that may substantially influence the final conclusion of the study. There is no unique or gold-standard procedure for performing a high-quality benchmark study on a given set of methods for a given question (hence the usefulness of the previously mentioned meta-analyses of benchmark studies). Computational scientists must thus redouble efforts in developing appropriate designs and reporting strategies for their comparison studies [2, 8], with the twin goals of more balanced reporting of new methods and an increase in the quality of neutral comparison studies.

In the meantime, scientists reading papers on new methods should keep in mind that this literature is potentially strongly biased. As our parents likely told us, newer is not *always* better.

## Supplementary Information

**Additional file 1** Supplementary information. This document includes the following supplementary information: supplementary methods, supplementary figures, supplementary tables, supplementary reference list.

**Additional file 2** Data set (excel file). The excel data file data_set_of_extracted_data_Buchka_et_al.xlsx contains the data from our bibliographical survey.

**Additional file 3** Review history.

## Data Availability

Data and R codes to reproduce the results are available from https://github.com/StefanBuchka/Papers[25]. Declarations
