# Supplementary material for: On the optimistic performance evaluation of newly introduced bioinformatic methods
Source: Genome Biol. 2021 May 11;22:152. doi: 10.1186/s13059-021-02365-4 (PMC8111726; doi:10.1186/s13059-021-02365-4)
Supplement: Supplementary file 1 — Additional file 1 Supplementary information. This document includes the following supplementary information: supplementary methods, supplementary figures, supplementary tables, supplementary reference list. [file 13059_2021_2365_MOESM1_ESM.pdf]

Supplement: On the optimistic performance evaluation of newly introduced bioinformatic methods

Stefan Buchka, Alexander Hapfelmeier, Paul P. Gardner, Rory Wilson, Anne-Laure Boulesteix

Date: 28.10.2020

Supplementary Methods

Selection of studies

To select relevant studies on HumanMethylation450 BeadChip normalization methods a nonsystematic search using the PubMed database was performed. The wording of the request is shown in Supplementary Figure 1, the flowchart of the process is shown in Supplementary Figure 2, and the list of chosen publications is shown in Supplementary List 1.

In addition to the selected publications from the search, two additional relevant articles were included in our study (Supplementary Figure 2, Supplementary List 1).

The inclusion criteria were:

- The studies were conducted in 2008 or later.
- The full text and the supplement section of the studies were available free of charge at the LMU in Munich.
- The studies used data of the Illumina Human Methylation450K BeadChip microarray or its successor the Illumina Human MethylationEPIC BeadChip microarray.
- The studies included comparisons and performance metrics of the relevant normalization methods.

The data extracted from the included papers are found in Supplementary Table 1.

Comparison of normalization methods

Our goal was to determine if papers introducing a method tend to be optimistic with regard to this method's performance in comparison to existing methods. For a given pair of methods, two types of comparisons were examined: a comparison in the paper introducing the newer of the two methods (*type-a* paper); and comparisons in later neutral papers, ie papers that are introducing neither of the two methods, and written by authors assumed to be unbiased (t*ype-b* papers). If optimistic bias is at work, *type-a* papers will tend to be more favourable to the method they are introducing than *type-b* papers are to the same method.

Specifically, for a given pair of methods for which a *type-a* paper exists and one or more *type-b* papers, we determined whether the *type-a* paper assesses the newly introduced method as better than its pair-partner, and whether the *type-b* papers also do so.

As our primary result, we examined the proportion of *type-a* papers evaluating the newly introduced method as better than its partner and the proportion of *type-b* papers also doing so, over all *type-a* and *type-b* papers examined.

If more than one method was introduced in a paper, then these pairs of simultaneously introduced methods were excluded from the analysis.

Determination of the "Better" Method

Analysis 1:

Our first analysis examined *type-a* papers and *type-b* papers as a whole, ie for each paper we determined one overall "better" method from the given pair. Most of the selected studies demonstrated the performance of the normalization methods in several graphical figures or performance metrics. We thus defined each such figure or metric as one "substudy" of the paper. Each substudy implies a ranking of all methods examined in the substudy, ie from best to worst determined through either numeric values or visual inspection by our rater. In the case of rank equality within a substudy, each method received the same rank as determined by a fractional ranking system: the best rank value of the equally ranked normalization methods, adding one divided by the number of equally performing methods. For example, methods A and

B are together the best ones. Method C is worse. The ranking would be A: 1.5, B: 1.5, and C: 3.

An overall determination of the "better" method of the pair for the paper was determined by examining the rankings of methods for all substudies within the paper: the "better" method was defined as that which had the lowest sum of rankings across these examined substudies.

In some cases, authors presented an overall ranking of the methods presented in their paper. If it was not possible to extract the results to reproduce their ranking, we used the ranking order provided by the authors to determine "better" methods.

In analysis 1, we only consider papers for which each substudy ranks every method examined in the paper (i.e., "complete substudies").

Analysis 2:

In our second analysis, we considered all substudies as separate entities/papers, ie we ignored that they group together in clusters (the original papers in which they appear). A method was considered the "better" method if it was ranked higher than the other within the specific substudy. Again, the proportion of *type-a* papers (in this case *type-a* substudies) ranking the newly introduced method as better than better than its partner and the proportion of *type-b* papers (here *type-b* substudies) also doing so were compared.

Some notes on evaluation of ranks within substudies:

1) If a performance metric is based on several samples of the same data set, the averages of the results across all samples for each method were calculated and used to determine the ranks.
2) The 450K BeadChip involves two types of probes (probe-type I and probe-type II), each interrogating different methylation sites. Often, the performances of the normalization methods for probe-type I and probe-type II were determined separately. In this case we averaged the rankings over the two probe types to determine a final rank for the normalization method.
3) Performance measurements based on specific CpG islands were omitted, as these measurements are very specific for each site/island and were not

considered representative of the overall performance of the examined normalization methods.

4) Non-readable figures and comparisons of normalization methods for which no ranking could be made were excluded.

The data extracted from the included papers are found in Supplementary Table 1.

Supplementary Figures

(DNA methylation data OR HumanMethylation450 data OR HumanMethylationEPIC data OR 450k data) AND (450k array OR BeadChip assay OR illumina OR microarray OR Infinium) AND (Normalization OR preprocessing OR pre-processing OR bias correction OR processing)

Supplementary Figure 1: Query of the PubMed literature research

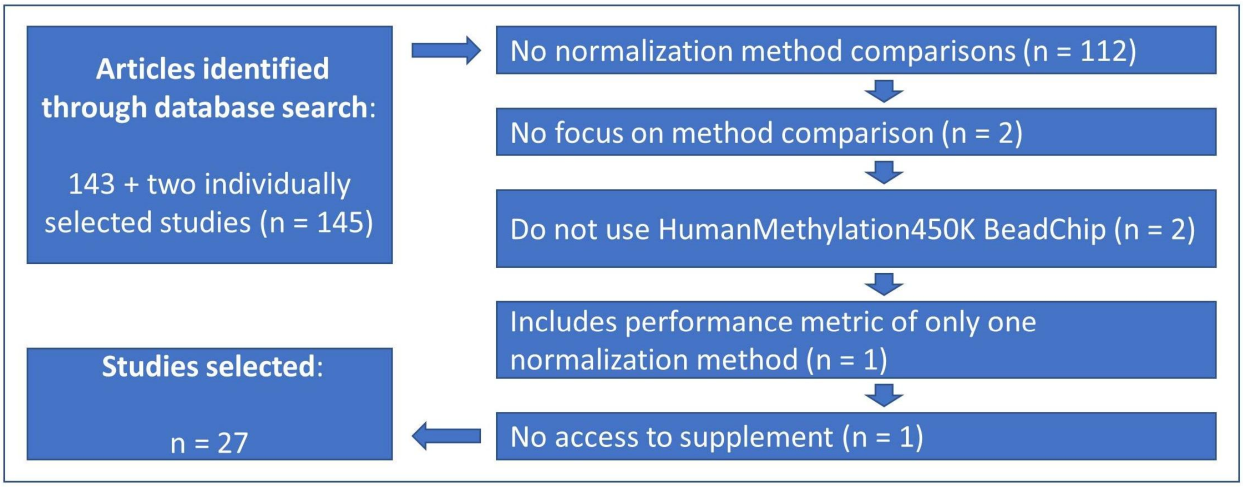

Supplementary Figure 2: Flowchart of the publication selection process

Supplementary Tables

Supplementary Table 1: Method comparison data extracted from the studies and used in the analyses

Presented are the data extracted from each paper selected for this study. Each row presents one method from one substudy, where a substudy is a comparison between preprocessing methods within the paper. The table is found in the external file data_set_of_extracted_data_Buchka_et_al.xlsx

Column name	Explanation
paper	Name of the paper in which the substudy is found.
PMID	PubMed ID of the paper
year	Year of publication of the paper
authors	First authors of the paper
introduced_method	The name of the method introduced in the paper, if any. If more than one method is introduced in the paper, the “better” method (as defined by the original authors) is listed here.
nb_methods_paper	Number of methods compared in the paper
nb_methods_substudy	Number of methods compared in the substudy
nb_datasets_paper	Number of datasets used in the paper
nb_datasets_substudy	Number of datasets used in the substudy
dataset_names	Name(s) of the dataset(s) used in the substudy
metric	Comparison metric used in the substudy

nb_metrics_substudy	Number of comparison methods used in the substudy; Normally=1; >1 indicates the results could not be reconstructed and the authors' overall rank (which involved more than 1 metric) was extracted
method	Name of the method
method_in_paper_as_named	If "y", the method in the paper has the same name as that given in the column "method". If blank, then the method in the paper has another name (given in the "Name_of_method_in_paper" column).
name_of_method_in_paper	If the method has a name in the paper different to that given in the "method" column, that name is given here.
rank	Rank of the method in the substudy
comment	Any additional comments

Supplementary Lists

Supplementary List 1: Selected publications

This list shows the publications selected for this study based on the query of Supplementary Figure 1 and the selection process of Supplementary Figure 2. The two additional included studies (i.e., beyond those resulting from the PubMed search) are shown in bold letters.

1 Aryee, M. J. et al. Minfi: a flexible and comprehensive Bioconductor package for the analysis of Infinium DNA methylation microarrays. Bioinformatics 30, 1363-1369, doi:10.1093/bioinformatics/btu049 (2014).

2 Cazaly, E. et al. Comparison of pre-processing methodologies for Illumina 450k methylation array data in familial analyses. Clin Epigenetics 8, 75, doi:10.1186/s13148-016-0241-2 (2016).

3 Dedeurwaerder, S. et al. A comprehensive overview of Infinium HumanMethylation450 data processing. Brief Bioinform 15, 929-941, doi:10.1093/bib/bbt054 (2014).

4 **Dedeurwaerder, S. et al. Evaluation of the Infinium Methylation 450K technology. Epigenomics 3, 771-784, doi:10.2217/epi.11.105 (2011).**

5 Fortin, J. P. et al. Functional normalization of 450k methylation array data improves replication in large cancer studies. Genome Biol 15, 503, doi:10.1186/s13059-014-0503-2 (2014).

6 Fortin, J. P., Triche, T. J., Jr. & Hansen, K. D. Preprocessing, normalization and integration of the Illumina HumanMethylationEPIC array with minfi. Bioinformatics 33, 558-560, doi:10.1093/bioinformatics/btw691 (2017).

7 Heiss, J. A. & Brenner, H. Between-array normalization for 450K data. Front Genet 6, 92, doi:10.3389/fgene.2015.00092 (2015).

8 Lehne, B. et al. A coherent approach for analysis of the Illumina HumanMethylation450 BeadChip improves data quality and performance in epigenome-wide association studies. Genome Biol 16, 37, doi:10.1186/s13059-015-0600-x (2015).

9 Liu, J. & Siegmund, K. D. An evaluation of processing methods for HumanMethylation450 BeadChip data. BMC Genomics 17, 469, doi:10.1186/s12864-016-2819-7 (2016).

10 **Maksimovic, J., Gordon, L. & Oshlack, A. SWAN: Subset-quantile within array normalization for illumina infinium HumanMethylation450 BeadChips. Genome Biol 13, R44, doi:10.1186/gb-2012-13-6-r44 (2012).**

11 Marabita, F. et al. An evaluation of analysis pipelines for DNA methylation profiling using the Illumina HumanMethylation450 BeadChip platform. Epigenetics 8, 333-346, doi:10.4161/epi.24008 (2013).

12 Niu, L., Xu, Z. & Taylor, J. A. RCP: a novel probe design bias correction method for Illumina Methylation BeadChip. Bioinformatics 32, 2659-2663, doi:10.1093/bioinformatics/btw285 (2016).

13 Pan, H. et al. Measuring the methylome in clinical samples: improved processing of the Infinium Human Methylation450 BeadChip Array. Epigenetics 7, 1173-1187, doi:10.4161/epi.22102 (2012).

14 Pidsley, R. et al. A data-driven approach to preprocessing Illumina 450K methylation array data. BMC Genomics 14, 293, doi:10.1186/1471-2164-14-293 (2013).

15 Shiah, Y. J., Fraser, M., Bristow, R. G. & Boutros, P. C. Comparison of pre-processing methods for Infinium HumanMethylation450 BeadChip array. Bioinformatics 33, 3151-3157, doi:10.1093/bioinformatics/btx372 (2017).

16 Teschendorff, A. E. et al. A beta-mixture quantile normalization method for correcting probe design bias in Illumina Infinium 450 k DNA methylation data. Bioinformatics 29, 189-196, doi:10.1093/bioinformatics/bts680 (2013).

17 Touleimat, N. & Tost, J. Complete pipeline for Infinium((R)) Human Methylation 450K BeadChip data processing using subset quantile normalization for accurate DNA methylation estimation. Epigenomics 4, 325-341, doi:10.2217/epi.12.21 (2012).

18 Triche, T. J., Jr., Weisenberger, D. J., Van Den Berg, D., Laird, P. W. & Siegmund, K. D. Low-level processing of Illumina Infinium DNA Methylation BeadArrays. Nucleic Acids Res 41, e90, doi:10.1093/nar/gkt090 (2013).

19 Wang, T. et al. A systematic study of normalization methods for Infinium 450K methylation data using whole-genome bisulfite sequencing data. Epigenetics 10, 662-669, doi:10.1080/15592294.2015.1057384 (2015).

20 Wang, Z., Wu, X. & Wang, Y. A framework for analyzing DNA methylation data from Illumina Infinium HumanMethylation450 BeadChip. BMC Bioinformatics 19, 115, doi:10.1186/s12859-018-2096-3 (2018).

21 Wu, M. C. et al. A systematic assessment of normalization approaches for the Infinium 450K methylation platform. Epigenetics 9, 318-329, doi:10.4161/epi.27119 (2014).

22 Yousefi, P. et al. Considerations for normalization of DNA methylation data by Illumina 450K BeadChip assay in population studies. Epigenetics 8, 1141-1152, doi:10.4161/epi.26037 (2013).

23 Bady, P., Delorenzi, M. & Hegi, M. E. Sensitivity Analysis of the MGMT-STP27 Model and Impact of Genetic and Epigenetic Context to Predict the MGMT

Methylation Status in Gliomas and Other Tumors. The Journal of molecular diagnostics : JMD 18, 350-361, doi:10.1016/j.jmoldx.2015.11.009 (2016).
24 McEwen, L. M. et al. Systematic evaluation of DNA methylation age estimation with common preprocessing methods and the Infinium MethylationEPIC BeadChip array. Clin Epigenetics 10, 123, doi:10.1186/s13148-018-0556-2 (2018).
25 Perrier, F. et al. Identifying and correcting epigenetics measurements for systematic sources of variation. Clin Epigenetics 10, 38, doi:10.1186/s13148-018-0471-6 (2018).
26 Xu, Z., Langie, S. A., De Boever, P., Taylor, J. A. & Niu, L. RELIC: a novel dye-bias correction method for Illumina Methylation BeadChip. BMC Genomics 18, 4, doi:10.1186/s12864-016-3426-3 (2017).
27 Xu, Z., Niu, L., Li, L. & Taylor, J. A. ENmix: a novel background correction method for Illumina HumanMethylation450 BeadChip. Nucleic Acids Res 44, e20, doi:10.1093/nar/gkv907 (2016).
