# Supplementary material for: On the optimistic performance evaluation of newly introduced bioinformatic methods
Source: Genome Biol. 2021 May 11;22:152. doi: 10.1186/s13059-021-02365-4 (PMC8111726; doi:10.1186/s13059-021-02365-4)
Supplement: Supplementary file 3 — Additional file 3 Review history. [file 13059_2021_2365_MOESM3_ESM.docx]

Review History

**First round of review**

**Reviewer 1**

**Are you able to assess all statistics in the manuscript, including the appropriateness of statistical tests used?**

There are no statistics in the manuscript.

**Comments to author:**

First I want to acknowledge that I really like the terms -- optimistic performance

Major comments:

1 -- In the first paragraph, the authors mention "lack of guidance". It would be nice to expand on what times of guidance is needed

2 -- The authors talk about publication bias. A more common term of "self-assessment trap". Consider citing: https://www.embopress.org/doi/full/10.1038/msb.2011.70 and https://www.nature.com/articles/s41592-020-0768-1?platform=oscar&draft=collection

3 -- Authors mention fixing bugs: It is not clear how this relates to the topic benign discussed (3rd paragraph)

4 -- Authors mention the failure of the original method to overperforms other methods when evaluated by benchmarking studies. It would be nice also to discuss that this can be due to different datasets being used. See an example of error correction methods, where the methods were evaluated on type of datasets not envisioned by authors of the software tools: https://genomebiology.biomedcentral.com/articles/10.1186/s13059-020-01988-3

5 -- Why author performs a pairwise comparison, and not for example triplets. The explanation needs to be provided

6 -- It is in general very hard to understand how the tools are compared. Are they compare across the original papers, or within. Are more clear explanation needs to be elaborated.

7 -- It very confusing the use of (a) (b) and (i) (ii) etc. And since authors refer to this as "in the first analysis". It is very hard to follow. The structure of the paper needs to be improved

8 -- Authors mention that new methods need to be evaluated in a balanced and transparent manner. How it is possible to achieve?

9 -- the last sentence sounds very informal. Please check with the editor if the language is appropriate.

10 -- x-axis . It is not clear what the abbreviation of the x-axis means

Minor comments:

1 -- In the first paragraph, the authors say that "strong contrast". It is unclear what the contract is

2 -- Please explain what is the meta-analysis of benchmarking studies. Is it to combine results of various studies?

3 -- The authors mentioned "neutral and blinded". Are those studies which are competition-based, where the gold standard is hidden from researchers running their tools?

4 -- "topical class". A strange choice of the term

5-- Figure: What means Number of comparisons? Is it a number of samples used to compare tools?

6 -- This is a sentence containing "evaluation of blurred graphical results" that needs to be improved for clarity. In a current form, it is hard to follow.

**Reviewer 2**

**Are you able to assess all statistics in the manuscript, including the appropriateness of statistical tests used?**

Yes, and I have assessed the statistics in my report.

**Comments to author:**

On the optimistic performance evaluation of newly introduced bioinformatic methods

Authors: Buchka, Hapfelmeier, Gardner, Wilson, Boulesteix

The authors consider the question: "are methods papers overoptimistic when introducing a new method" and presents a qualitative analysis of a specific field: normalization of DNA methylation microarrays.

The data presented are anecdotal in nature in the sense that they cover a single specific field in computational biology. Nevertheless, the study design is excellent and the subject matter makes it interesting to any methods developer and potentially to some methods users. Personally, I have definitely debated this question over the years with colleagues and it is great to finally have some evidence for the expectation of inherent optimism.

The study itself has a great design and I have no major comments on this. My main issue is with the conclusion and recommendations which I believe should be expanded a bit, as I try to explain below.

Following the description of their observations, the authors state "These observations highlight a disturbing problem in the computational literature." and follow with 3 recommendations. The authors' thinking is not clear to me.

What is the problem exact and whose problem is it? Is the problem that methods authors are optimistic? Is that the methods authors' problem? Is it the users problem? Is it the journals problem? Depending on what the problem is and whose problem it is, different solutions emerge. It is not at all clear to me that the recommendations the authors put forth really address the problem. This is of course a subjective discussion. What I am looking for here are more detailed arguments so I can understand Buchka et al's perspective on these questions. I get the feeling I might disagree with some of their opinions (which is fair), but I would like to know what their stance is.

For example, if the problem is that methods authors should not be so optimistic, why does benchmarking papers help? After all, benchmarking happens after method paper! Or is the issue rather that the community needs better recommendations. In that case, should we change anything about individual method papers? Or is it the case that what makes a good/best method is highly dependent on the question and the specific dataset at hand. In that case, perhaps the solution is to educate users that selecting the "best" method is challenging and actually requires some level of expertise; in other words, the easy answer does not exist. I believe all of these scenarios are relevant, but the problems and solutions are different. In short, what ideal state of the world does Buchka et al imagine, and do their suggestions actually move us toward this state?

I want to comment on the high weight Buchka et al puts on independent benchmark papers. This makes sense in the context of the question they examine - one can hardly argue that benchmark papers are biased (in a personal sense). But for judging which method is the best (which seems partly the problem Buchka et al put forth), benchmarking papers are only as good as the measures they use. And sometimes these measures are not good, at least in my opinion. Indeed, figuring out the best method is extremely hard; as an example, it is interesting that Buchka et al completely avoids answering this question despite having read such a significant part of the literature (I don't fault them for avoid the question, but I find it illustrates the complexity). This is relevant in terms of their 1st recommendation, where they implicitly argue that benchmarking papers are inherently superior (I am not questioning that they are useful, I am questioning their inherent superiority). In fact, the methylation array literature is a good example of this, where the comment evaluation criteria of reducing technical variance has been questions as irrelevant by Fortin et al (2014 Genome Biology) and has nevertheless been used in benchmarking papers such as Shiah et al (2017 Bioinformatics). An interesting (perhaps impossible) study would be to assess to which degree different benchmarking papers actually agree in their conclusions and - if they do not agree - what does this tell us?

In my reading of the current lay of the land, there is quite a lot of appreciation for benchmark studies (perhaps even too much). This was not the case 10 years ago, but I believe this has changed.

Minor issues.

(a) Funnorm and Funnormwithnoob was introduced in the same paper so I don't understand which one is newest as per the caption of Figure 1.

(b) The authors do not comment on "effect size". In some comparisons it is pretty clear that method A is only a tiny bit better than method B, but the authors just consider "better". I acknowledge the complexity of considering effect size in their comparison, but I think a comment is needed.

**Authors Response**

**Point-by-point responses to the reviewers’ comments:**

*We are grateful to the reviewers for their careful evaluation of our submission, their valuable comments and their helpful suggestions, which contributed to a significant improvement in the quality of the paper. All comments have been addressed, and the manuscript has been revised accordingly. In the revised manuscript, changes from the original version are highlighted in red.*

*Since one of the reviewers mentioned that the structure is not clear enough, we introduced new headings. However, we understand that headings are not allowed in short papers according to the instructions to authors. We are open to various solutions depending on the editors’ preference (e.g., giving up the headings or making a full paper out of the study).*

Reviewer 1

Major comments:

1.	In the first paragraph, the authors mention ”lack of guidance”. It would be nice to expand on what times of guidance is needed

*We added a sentence in the first paragraph to clarify this point:*

*“It is not clear how new methods and their performances should be described and how studies comparing the performances of methods should be designed.”*

2.	The authors talk about publication bias. A more common term of ”self-assessment trap”. Consider citing: https://www.embopress.org/doi/full/10.1038/msb.2011. 70 and https://www.nature.com/articles/s41592-020-0768-1?platform=oscar& draft=collection

*Thanks for mentioning these relevant references. We now discuss them:*

*“The term “self-assessment trap” has also be used to describe these mechanisms (Norel et al., 2011; Marx, 2020)”*

*“It is however much lower than the 94.7% observed in the first analysis, which is in agreement with Norel et al.’s claim that “when the number of performance metricsis larger than two, most methods fail to be the best in all categories assessed”*

*“or performance metrics (Norel et al., 2011)”.*

3.	Authors mention fixing bugs: It is not clear how this relates to the topic benign discussed (3rd paragraph)

*We now better explain this idea.*

*“For example, if a new method yields a noticeably bad result in one analysis. The authors are likely to look for a potential bug, while they would accept the bad result—without further investigation—if observed for a competing method.”*

4.	Authors mention the failure of the original method to overperforms other methods when evaluated by benchmarking studies. It would be nice also to discuss that this can be due to different datasets being used. See an example of error correction meth¬ods, where the methods were evaluated on type of datasets not envisioned by authors of the software tools: https://genomebiology.biomedcentral.com/articles/10.1186/s13059-020-01988-3

*We thank the reviewer for pointing out the issues with different types of datasets used, especially regarding unanticipated types. We have added the following sentences:*

*“And although this bias weakens with a greater number of datasets used for fitting and evaluation, another problem may be that authors test their method on datasets from contexts with which they are familiar, and failing to outline this bias in the limitations. Previous studies have indicated that performance evaluation can highly depend on the type of datasets examined (Mitchell et al., 2020), a source of variation perhaps revealed in later studies involving types of datasets unanticipated by the original method’s authors.”*

5.	Why author performs a pairwise comparison, and not for example triplets. The ex-planation needs to be provided.

*See our answer to point 6.*

6.	It is in general very hard to understand how the tools are compared. Are they com¬pare across the original papers, or within. Are more clear explanation needs to be elaborated.

*We apologize that the design of our study was not explained clearly enough. We consider pairs of methods that have been compared in both the paper introducing the newer of the two and in subsequent papers that were (assumed to be) “neutral” with respect to the pair. We look whether the newer method of the two was ranked better in both types of papers. Considering triplets of methods would make everything much more complicated, and there would be many fewer triplets than pairs compared in the same paper. We now better explain the design of our study and illustrate it through a new figure. We also make clearer reference to the supplement, which details all methods in full.*

*“This principle is schematically illustrated in Figure 1. Imagine that a method “B” is introduced in the literature some time after a method “A” had been introduced. In the paper presenting method B, the authors compare it to method A (typically, they find that B is better than A). This is what we call a non-neutral comparison because the authors are interested in demonstrating the superiority of method B. Some time later, a paper suggesting another method, “C”, is published. The authors compare C to existing methods A and B, which implies that this study also compares B to A, although this latter comparison is not the paper’s focus. This study is assumed to be neutral with respect to A and B, exactly as the later study termed a “neutral benchmark study” in Figure 1.*

*In the simplified scenario of Figure 1, we have one non-neutral study and two neutral studies, all comparing A and B. In the terminology commonly used in the debate on the replication crisis, the former could be denoted as an “original study” and the latter two as “replication studies”. Of course, in general there may be any number of neutral studies, not just two. Most importantly, in our survey we did not focus on a specific pair of methods, but looked for all pairs that were compared in the non-neutral study introducing the newer one and at least one subsequent neutral study.”*

7. It very confusing the use of (a) (b) and (i) (ii) etc. And since authors refer to this as ”in the first analysis”. It is very hard to follow. The structure of the paper needs to be improved

*We originally introduced the notations (a) and (b) to avoid repetition, but have now removed them to make the paper more understandable. We also removed (i), (ii), (iii) from the last paragraph, which has been completely restructured. Regarding the structure of the paper, we introduced headings to make it clearer.*

8.	Authors mention that new methods need to be evaluated in a balanced and trans-parent manner. How it is possible to achieve?

*Good point! We added an explanation:*

*“They would not cherry-pick their best results, for example across a large set of results obtained through different configurations of datasets, simulation scenarios, parameter settings or performance metrics, while sweeping the other results—those making the new method look less impressive—under the carpet... Editors and reviewers should become more tolerant towards methods that are not reported to perform best universally...”*

9.	the last sentence sounds very informal. Please check with the editor if the language is appropriate.

*This was intentional. We thought it would be nice to end the paper with a catchy sentence. But of course we can change that if this approach is not appreciated.*

10.	x-axis. It is not clear what the abbreviation of the x-axis means

*We amended the sentence “the x-axis shows which two methods are being compared” into:*

*“the x-axis shows which two methods are being compared (see the additional files 1 and 2 for the references of the methods, abbreviations and any aliases used in the paper).”*

Minor comments:

1. In the first paragraph, the authors say that ”strong contrast”. It is unclear what the contract is

*The absence of guidance in computational research is in contrast to the intensive decades-long discussions in the biomedical literature on the design of other types of health science studies and the resultant reporting guidelines developed, such as those for observational studies. We added the phrase “and guidance documents” af¬ter ”guidelines” and the word “numerous” to make clearer which contrast we mean. “This absence of guidance is in strong contrast to the intensive decades-long dis-cussions in the biomedical literature on the design of other types of health science studies and the numerous resultant reporting guidelines and guidance documents developed...”*

2.	Please explain what is the meta-analysis of benchmarking studies. Is it to combine results of various studies?

*Yes, the reviewer is correct. We now state that more clearly.*

*“(i.e. studies combining the results of several benchmark studies)”*

3.	The authors mentioned ”neutral and blinded”. Are those studies which are competition-based, where the gold standard is hidden from researchers running their tools?

*We now mention challenges explicitly. Regarding blinding, we refer to benchmark studies with some sort of blinding procedure aiming at reducing the bias. Although it would have been interesting to do so, we think that explaining this procedure in detail would go beyond the scope of this short report and thus refer to the paper by Kreutz and colleagues. The sentence now reads:*

*“...(i.e. systematic studies comparing the behaviours and performances of computa-tional methods) ... —especially those that are neutral (Boulesteix et al., 2013), and those that incorporate a blinding procedure (Kreutz et al., 2020), community com-putational challenges and meta-analyses of benchmarking studies (Gardner et al., 2019) (i.e. studies combining the results of several benchmark studies) are crucial in providing guidance for the methods’ potential users (Robinson and Vitek, 2019).”*

4.	”topical class”. A strange choice of the term

*We have removed the confusing term from the manuscript.*

5.	Figure: What means Number of comparisons? Is it a number of samples used to compare tools?

*The number of comparisons refers to the number of papers (for the first analysis, which takes papers as units) or to the number of substudies (for the second analysis, which takes substudies as units). This is now stated explicitly in the legend of the figure.*

6.	This is a sentence containing ”evaluation of blurred graphical results” that needs to be improved for clarity. In a current form, it is hard to follow.

*We have rephrased the description as follows and hope it is now clearer:*

*The evaluator extracting the data could obviously not be blinded to the type of paper (non-neutral or neutral). This lack of blindness could have slightly distorted our results, as the evaluator’s expectation was that new methods would tend to be optimistically rated in the papers introducing them. This expectation might have affected, for example, his (partly subjective) evaluation of blurred or ambiguous graphical results within the papers being evaluated.*

Reviewer 2

The authors consider the question: ”are methods papers overoptimistic when introducing a new method” and presents a qualitative analysis of a specific field: normalization of DNA methylation microarrays. The data presented are anecdotal in nature in the sense that they cover a single specific field in computational biology. Nevertheless, the study design is excellent and the subject matter makes it interesting to any methods developer and potentially to some methods users. Personally, I have definitely debated this question over the years with colleagues and it is great to finally have some evidence for the expectation of inherent optimism.

*Thanks for this positive feedback!*

The study itself has a great design and I have no major comments on this. My main issue is with the conclusion and recommendations which I believe should be expanded a bit, as I try to explain below. Following the description of their observations, the authors state ”These observations highlight a disturbing problem in the computational literature.” and follow with 3 recommendations. The authors’ thinking is not clear to me. What is the problem exact and whose problem is it? Is the problem that methods authors are op¬timistic? Is that the methods authors’ problem? Is it the users problem? Is it the journals problem? Depending on what the problem is and whose problem it is, different solutions emerge. It is not at all clear to me that the recommendations the authors put forth really address the problem. This is of course a subjective discussion. What I am looking for here are more detailed arguments so I can understand Buchka et al’s perspective on these questions. I get the feeling I might disagree with some of their opinions (which is fair), but I would like to know what their stance is.

*We extended, rewrote and restructured the last paragraphs of the paper to address these important issues raised by the reviewer.*

For example, if the problem is that methods authors should not be so optimistic, why does benchmarking papers help? After all, benchmarking happens after method paper! Or is the issue rather that the community needs better recommendations. In that case, should we change anything about individual method papers?

*We addressed especially this point in the new version of the last paragraphs by explaining that we need both papers on new methods that are less biased and more neutral comparison studies.*

Or is it the case that what makes a good/best method is highly dependent on the question and the specific dataset at hand. In that case, perhaps the solution is to educate users that selecting the ”best” method is challenging and actually requires some level of expertise; in other words, the easy answer does not exist.

*We added this point to our discussion, thanks!*

*“Users of methods should be educated in the proper interpretation of benchmark studies: selecting methods requires expertise, and benchmark studies give a limited picture of the situation in a specific context perhaps not relevant to the given reader. For example, the choice of adequate performance measures is not easy. More generally, researchers conduct-ing benchmark studies have to make a myriad of design choices that may substantially influence the final conclusion of the study.”*

I believe all of these scenarios are relevant, but the problems and solutions are different. In short, what ideal state of the world does Buchka et al imagine, and do their suggestions actually move us toward this state? I want to comment on the high weight Buchka et al puts on independent benchmark papers. This makes sense in the context of the question they examine - one can hardly argue that benchmark papers are biased (in a personal sense). But for judging which method is the best (which seems partly the problem Buchka et al put forth), benchmarking papers are only as good as the measures they use. And sometimes these measures are not good, at least in my opinion. Indeed, figuring out the best method is extremely hard; as an example, it is interesting that Buchka et al completely avoids answering this question despite having read such a significant part of the literature (I don’t fault them for avoid the question, but I find it illustrates the complexity). This is relevant in terms of their 1st recommendation, where they implicitly argue that bench-marking papers are inherently superior (I am not questioning that they are useful, I am questioning their inherent superiority). In fact, the methylation array literature is a good example of this, where the comment evaluation criteria of reducing technical variance has been questions as irrelevant by Fortin et al (2014 Genome Biology) and has nevertheless been used in benchmarking papers such as Shiah et al (2017 Bioinformatics). An interest¬ing (perhaps impossible) study would be to assess to which degree different benchmarking papers actually agree in their conclusions and - if they do not agree - what does this tell us?

*We now also discuss this aspect. We essentially agree with the reviewer, but think that it is not a “limitation of benchmark studies” but a general limitation affecting all evalua¬tions of methods including those performed in papers on new methods. More precisely, we now mention as a limitation of the study that we did not assess the quality of the perfor¬mances measures:*

*“in particular the quality of the performance measures”*

*and also mention this point in the paragraph that recommends performing more neutral benchmark studies (and refer to the example of the reduction of technical variation inves-tigated by Fortin and colleagues):*

*“For example, the choice of appropriate performance measures is not easy; in the context of methylation data analysis, see the case of evaluation criteria related to the reduction of tech-nical variation, which are widely used but of questionable relevance (Fortin et al.,2014)”.*

In my reading of the current lay of the land, there is quite a lot of appreciation for 7

benchmark studies (perhaps even too much). This was not the case 10 years ago, but I believe this has changed.

*We agree with the reviewer and added a comment to underline this point:*

*“as some journals including Genome Biology (Robinson and Vitek, 2019) have already started to do in the last few years”.*

Minor issues.

(a)	Funnorm and Funnormwithnoob was introduced in the same paper so I don’t under¬stand which one is newest as per the caption of Figure 1.

*The reviewer is right. We agree that it made poor sense to include this pair in the analysis—and in fact it does not fit our criterion of “new” vs “existing” methods— and have removed it, and double-checked the data for any other potentially similar instances. The figure and results have been updated accordingly, but have not changed in any substantial manner.*

(b)	The authors do not comment on ”effect size”. In some comparisons it is pretty clear that method A is only a tiny bit better than method B, but the authors just consider ”better”. I acknowledge the complexity of considering effect size in their comparison, but I think a comment is needed.

*Thanks for mentioning this important point. We added it to the limitations of the study:*

*“Also not taken into account in our study were the sizes of the differences between method performances: a method was considered either better or worse than the other, which obviously leads to a loss of information and precision.”*

**Second round of review**

**Reviewer 1**

All my comments were adequately addressed
